# *In situ* genetic correction of *F8* intron 22 inversion in hemophilia A patient-specific iPSCs

**DOI:** 10.1038/srep18865

**Published:** 2016-01-08

**Authors:** Yong Wu, Zhiqing Hu, Zhuo Li, Jialun Pang, Mai Feng, Xuyun Hu, Xiaolin Wang, Siyuan Lin-Peng, Bo Liu, Fangping Chen, Lingqian Wu, Desheng Liang

**Affiliations:** 1 State Key Laboratory of Medical Genetics, School of Life Sciences, Central South University, Changsha, Hunan, China; 2 Department of Hematology, Xiangya Hospital, Central South University, Changsha, Hunan, China; 3 Hunan Jiahui Genetics Hospital, Changsha, Hunan, China

## Abstract

Nearly half of severe Hemophilia A (HA) cases are caused by *F8* intron 22 inversion (Inv22). This 0.6-Mb inversion splits the 186-kb *F8* into two parts with opposite transcription directions. The inverted 5′ part (141 kb) preserves the first 22 exons that are driven by the intrinsic *F8* promoter, leading to a truncated *F8* transcript due to the lack of the last 627 bp coding sequence of exons 23–26. Here we describe an *in situ* genetic correction of Inv22 in patient-specific induced pluripotent stem cells (iPSCs). By using TALENs, the 627 bp sequence plus a polyA signal was precisely targeted at the junction of exon 22 and intron 22 *via* homologous recombination (HR) with high targeting efficiencies of 62.5% and 52.9%. The gene-corrected iPSCs retained a normal karyotype following removal of drug selection cassette using a Cre-LoxP system. Importantly, both *F8* transcription and FVIII secretion were rescued in the candidate cell types for HA gene therapy including endothelial cells (ECs) and mesenchymal stem cells (MSCs) derived from the gene-corrected iPSCs. This is the first report of an efficient *in situ* genetic correction of the large inversion mutation using a strategy of targeted gene addition.

Hemophilia A (HA) is an X-linked recessive congenital bleeding disorder with an occurrence of 1 in 5,000 male births, and affects nearly 80%–85% of patients with hemophilia^1^. HA is caused by a deficiency of the clotting factor VIII (FVIII), encoded by the factor VIII gene (*F8*)^2^,^3^. Affected males suffer from varying degrees of symptoms including intracranial hemorrhage, which might be lethal to patients with severe hemophilia. About 50%–60% of the HA patients are severe hemophiliac (<%1 of the normal clotting activity), characterized by recurrent spontaneous bleeding incidents.

Hemophilia has long been identified as an ideal target for gene therapy, and even a slight increase of clotting factor (>1%) in the blood can remarkably improve the quality of life for severe hemophilia patients. Although with a lower incidence of 1 in 30,000 male births, hemophilia B (HB) has been favored in gene therapy research and a long-term expression of therapeutic levels of FIX has been achieved by adeno-associated viral (AAV)-based gene therapy^4^. However, such a promising approach is not applicable for HA at the present stage, mainly owing to the size of the FVIII coding sequence that at 7 kb far exceeds the normal packaging capacity of AAV vectors.

*In situ* correction of the mutated gene is believed to be the ultimate gene therapy strategy for hemophilia, while needing more specialized technologies^5^. The corrected genes are still under the control of the endogenous promoter and other related regulatory elements, rather than forced ectopic expression of therapeutic genes that is widely used in hemophilia gene therapy research. The intron 22 inversion (Inv22) mutation of *F8* causes about 45% of severe HA cases. It is the result of intrachromosomal recombination between the nested gene A within intron 22 and either of the two additional copies of gene A lies 0.5 Mb telomeric to *F8*^6^. This 0.6 Mb inversion leads to a complete disruption of *F8* by splitting it into two parts (141 kb and 45 kb) that can be transcribed in opposite directions^7^. The 5′ part (141 kb) is inverted and preserves the *F8* promoter region. Interestingly, the coding sequence of the last four exons left in the 3′ part (45 kb) is only 627 bp, leading to a strategy of targeted addition of this 627 bp to the 5′ part to complement an *F8* transcript under the original promoter.

We report here an *in situ* genetic correction of the Inv22 mutation in HA patient-specific induced pluripotent stem cells (iPSCs) by using the transcription activator-like effector nucleases (TALENs), resulting in a rescue of both *F8* transcription and FVIII secretion in the endothelial cells (ECs) and mesenchymal stem cells (MSCs) derived from the gene-corrected iPSCs.

## Results

### Generation and Characterization of patient-specific iPSCs

**Figure 1:**
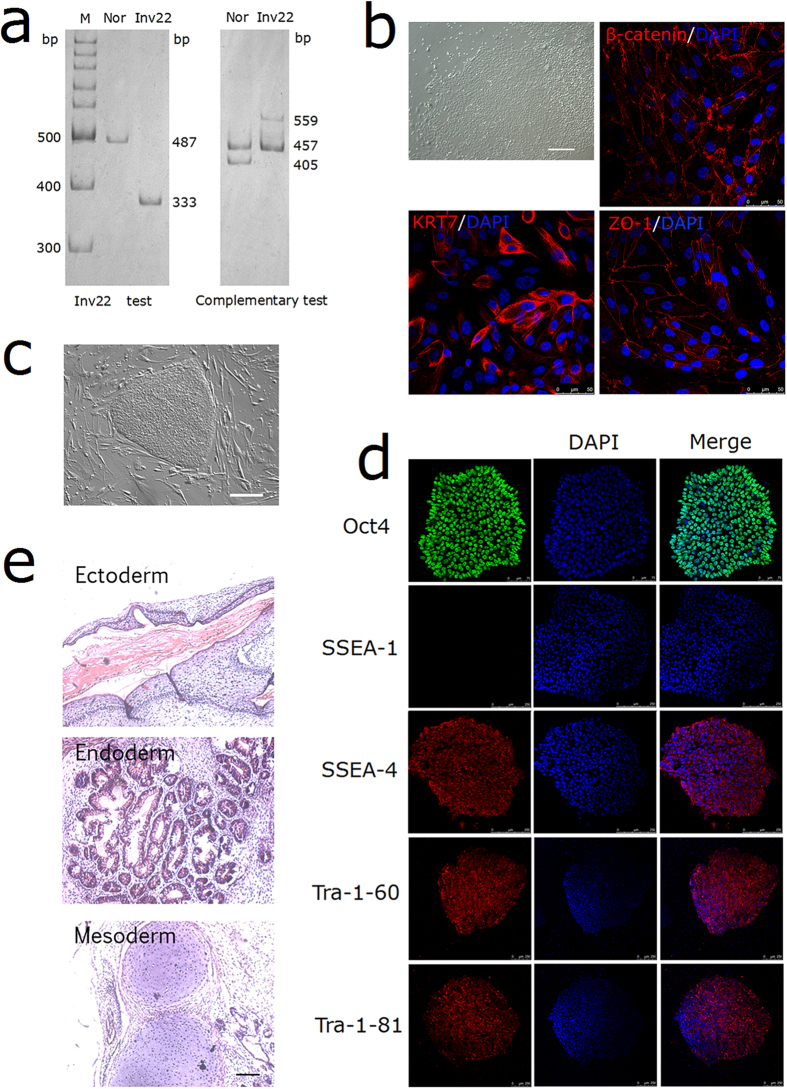
Genotyping PCRs and the generation of iPSCs. (**a**) Molecular diagnosis of *F8* intron 22 inversion using IS-PCR. Abbreviations: M indicates molecular markers; Nor, normal control; Inv22, intron 22 inversion. Both the Inv22 test and Complementary test results were cropped from the same gel, the full-length gel was presented in Supplementary Fig. S7a. (**b**) Morphology of the primary epithelial cells and immunostaining of β-catenin, KRT7 and ZO-1, DAPI was used to visualize the nucleus. (**c**) Brightfield of a representative iPSC clump on MEFs. (**d**) Immunostaining of iPSCs expressing markers for Oct 4, SSEA-4, Tra-1-60, Tra-1-81 and the differentiating marker SSEA-1, the DAPI staining indicates the total cell nucleus per field. (**e**) H&E staining of teratomas derived from immunodeficient mice injected with HA patient-specific iPSCs shows tissues representing all three embryonic germ layers: ectoderm (squamous epithelium), endoderm (respiratory epithelium), and mesoderm (cartilage). Scale bar represent 200 μm for (panels **b**,**c**,**e**).

Genomic DNA of a 51-year old male patient with severe HA was digested with BclI and ligated and used as template for IS-PCR^8^. A 333 bp fragment was detected in the inv22 diagnostic test, while a 559 bp and a 457 bp signals were detected in the complementary test that precludes the possibility of intron 22 deletion (Fig. 1a), indicating a diagnosis of the distal pattern of Inv22.

Although dermal fibroblast is the most common initial cell type used for iPSC reprogramming, invasive sampling should be avoided for hemophiliac patients. Therefore, we collected and expanded urine cells from urine sample of the patient. Small colonies formed as early as three days after seeding with cobblestone-like appearance (Fig. 1b). These cells grew quickly and expressed adherens junction marker β-catenin, epithelial marker intermediate filament keratin 7 (KRT7) and tight junction marker zonula occludens-protein 1 (ZO-1) (Fig. 1b). These results are consistent with those in previous papers about culture of human exfoliated epithelial cells from urinary tract and the renal tubular system^9^,^10^. Four reprogramming factors (Oct4, Sox2, Klf4 and c-Myc) were transduced into urine cells using retroviral vectors^9^. After continuous culture on MEFs, hESC-like clones emerged about 3 weeks later. Single clones were picked up and expanded for further characterization (Fig. 1c). To test whether the clones have the characteristics of human embryonic stem cell (hESC), we examined pluripotent markers expression *via* immunofluorescence. The clones expressed ESC transcription factor Oct4 and surface antigens stage-specific embryonic antigen (SSEA)-4, tumor-related antigen (TRA)-1-60 and TRA-1-81, while SSEA-1 expression could not be detected (Fig. 1d). To further evaluate the pluripotency *in vivo*, we injected the cells subcutaneously into immunocompromised mice. Two months later, the formed teratomas were harvested and sectioned. After stained with hematoxylin and eosin, tissues derived from the three embryonic germ layers were observed, including squamous epithelium (ectoderm), respiratory epithelium (endoderm) and cartilage (mesoderm) (Fig. 1e).

### Generation of TALENs

**Figure 2:**
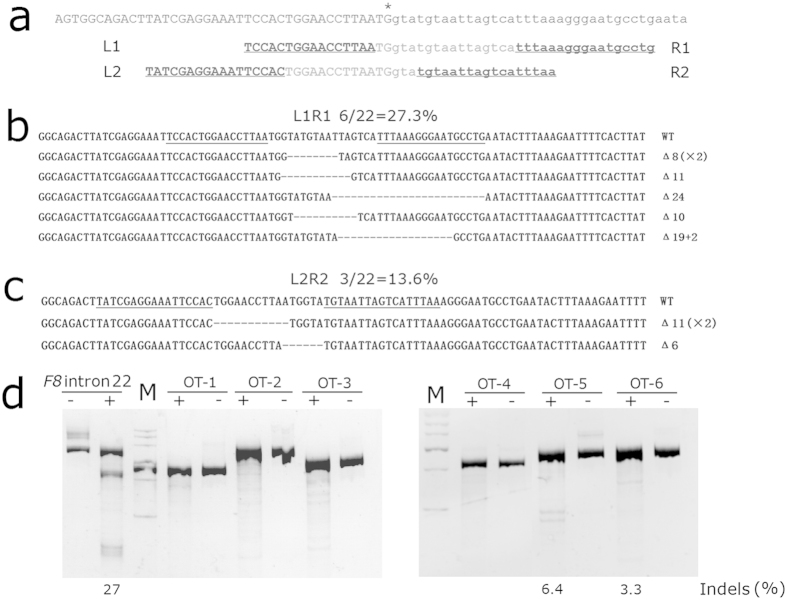
TALEN design and detecting of the indels. (**a**) The design of the two pairs of TALENs. Asterisk indicates the junction of exon 22 and intron 22. The underlined letters indicate the binding sites of the TALENs. (**b,c**) Sanger sequencing of the indels induced by each TALEN pair in HEK293T cells. Abbreviations: WT, wild type; △, deletion. (**d**) T7E1 assay of cutting activity of L1R1 at designed target site and six potential off-target (OT) sites. The percentage of indels was indicated. The gels represented the full-length gels.

TALENs can be engineered to recognize specific genomic sequence and induce double strand breaks (DSBs), which can stimulate targeted gene manipulation dramatically. We designed and constructed two pairs of TALENs (designated L1R1 and L2R2) precisely targeting at the junction of exon 22 and intron 22 of *F8* (Fig. 2a)^11^. Prior of gene targeting, we tested the cleavage activity of the TALENs in HEK293T cells. Each pair of the TALENs was transiently expressed in HEK293T cells, and then the genomic region encompassing the nuclease target site was PCR amplified, cloned and sequenced. The cleavage activity could be reflected by the percentage of insertions and/or deletions (indels) that are the characteristics of the non-homologous end joining (NHEJ), an error-prone DNA repair pathway by which the TALEN-mediated DSBs are mainly repaired. For each TALEN pair, 22 sequences were obtained through Sanger sequencing. Six and three mutated alleles were identified for L1R1 and L2R2 respectively, indicating the corresponding gene-disrupting rates of 27.3% and 13.6% (Fig. 2b,c). All 9 mutations are deletions, being consistent with the mutation signature of TALENs as reported previously^12^. We predicted the off-target sites of L1R1 using an online bioinformatics tool^13^. The DNA cleavage at six of the highly ranked potential targets was analyzed by T7E1 assay in HEK293T cells. Two of the sites showed detectable indels (6.4% and 3.3%). Although two other sites also showed weak digestion by T7E1, the banding pattern indicated that the mismatch was not at the predicted cleavage sites, the grayscale values of the bands were not used for calculating indels (Fig. 2d).

### Gene targeting and excision of PGK-Neo

**Figure 3:**
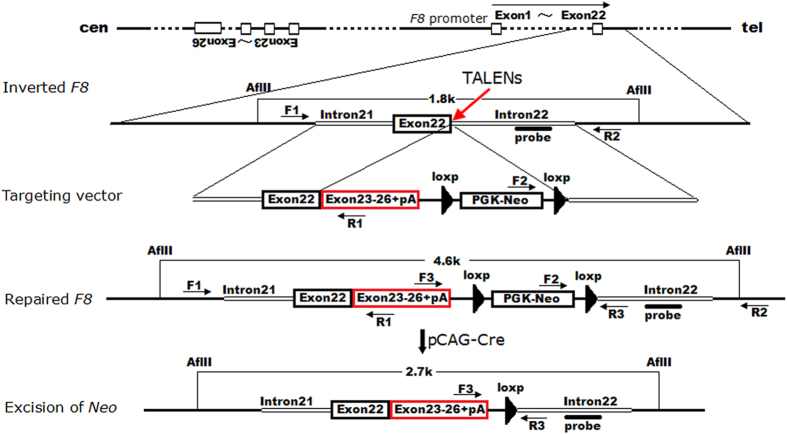
Schematic representation the *in situ* gene correction and the excision of PGK-Neo cassette. The targeting vector will integrate the coding sequence of exon 23–26, an SV40 polyA signal (pA) and a floxed PGK-Neo cassette at the junction of exon 22 and intron 22. TALENs were used to stimulate the homologous recombination. Primers F1/R1 and F2/R2 were used in PCR screening of the homologous integrants. Probe used in Southern blot was located in intron 22. Then the floxed PGK-Neo cassette was removed from the genome using a Cre-LoxP system. Primers F3 and R3 were used in the PCR screening of excision. All the sizes of the restriction fragments in Southern blot analysis were indicated.

**Figure 4:**
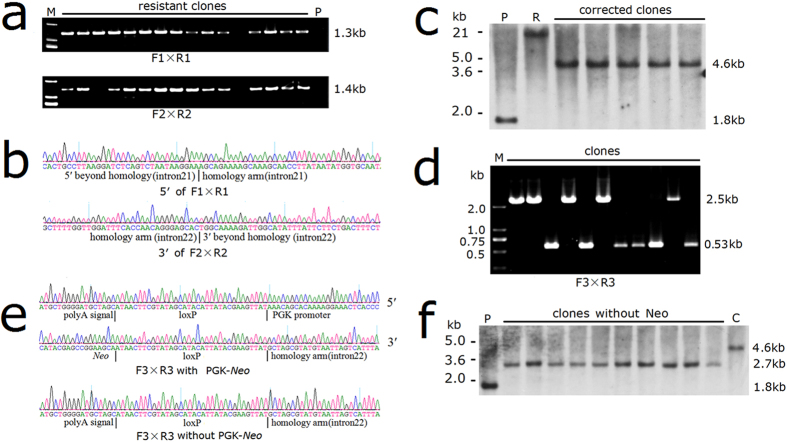
PCR and Southern blot analysis of the gene correction and the excision of PGK-Neo cassette. (**a**) PCR screening of the resistant clones in the first step of gene correction. The sizes of the PCR products for homologous recombinants were 1.3 kb and 1.4 kb using primers F1/R1 and F2/R2 respectively. Agarose gel results were cropped and the full-length gels were presented in Supplementary Fig. S7b. All the gels were run under the same condition (**b**) The PCR products were cloned and sequenced by Sanger sequencing. (**c**) The PCR-positive clones were confirmed by Southern blot analysis of the AflII digested genomic DNA. The blots were cropped and the full-length blots were presented in Supplementary Fig. S7c. (**d**) After being transfected by pCAG-cre, single clones were screened by PCR using primers F3 and R3. The gels represented the full-length gels. (**e**) The PCR products using F3 and R3 were cloned and sequenced by Sanger sequencing. (**f**) The PCR-positive clones were confirmed by Southern blot analysis. The blots were cropped and the full-length blots are presented in Supplementary Fig. 7d. Abbreviations: M, markers; P, the initial patient cells; R, randomly integrated clones; C, gene corrected clones with PGK-Neo cassette.

We constructed a donor vector containing the coding sequence of exons 23–26, an SV40 polyA signal and a floxed PGK-Neo cassette. These sequences were flanked by an 899-bp left homologous arm and a 909-bp right arm (Fig. 3). To perform gene targeting, TALENs and linearized donor vector were nucleofected into the patient-specific iPSCs. After two weeks of drug selection with G418, single cell colonies were individually picked up and expanded. These clones were initially PCR screened for targeted integration at both 5′ and 3′ site (Figs 3 and 4a). The PCR products were sequenced by Sanger sequencing (Fig. 4b). The PCR-positive clones were then confirmed by Southern blot analysis (Figs 3 and 4c). To test whether the donor vector could correct the *F8* mutation alone, 5 million of iPSCs were transfected by only the donor vector and totally 30 drug-resistant clones were obtained, whereas no clones were indentified as homologous recombinant. When L1R1 were co-transfected, as many as 568 resistant clones were obtained from 6 million of initial iPSCs. Sixteen clones were picked up and analyzed by PCR and Southern blotting, 10 of which were confirmed as targeted clones, indicating a targeting efficiency of 62.5%. Although L2R2 were less mutagenic than L1R1 as tested in HEK293T cells, high efficiency of 52.9% was achieved when co-transfected with the donor vector (Table 1).

For PGK-Neo cassette deletion, cells were trypsinized and nucleofected with pCAG-Cre (Addgene). After continuous culture, the emerged colonies were picked up and expanded for PCR and Southern blot analysis. In the two experiments we have conducted, Neo was removed in 31% (4/13) and 43% (9/21) of the single cell-derived clones respectively (Figs 3 and 4d–f). Two of the PGK-Neo deleted clones were designated 17-9 and 11-10 and were used in the following experiments.

We tested whether unwanted mutations were introduced during the reprogramming and targeting process. G-banded chromosomes were analyzed and no robust chromosomal aberration was found in corrected cells (Supplementary Fig. S1). Although a gain of 295,170 bp (nt. 25615710-25910879) in 22q11.23 was found during copy number variation (CNV) analysis of the initial urine cells, no further CNV was detected in iPSCs after genetic correction (Supplementary Fig. S2). After gene targeting, we sequenced the potential off-target sites of the TALENs predicted by the PROGNOS bioinformatic tool^13^. At all the potential off-target sites, no indels were found in the corrected clones 11-10 and 17-9 (Supplementary Fig. S4; Supplementary Table S2). Likewise, we predicted and sequenced the potential off-target sites of L2R2 and no indels were found at the sites in L2R2 stimulated clones too (Supplementary Fig. S5; Supplementary Table S2).

### FVIII expression in iPSCs and differentiated cells

**Figure 5:**
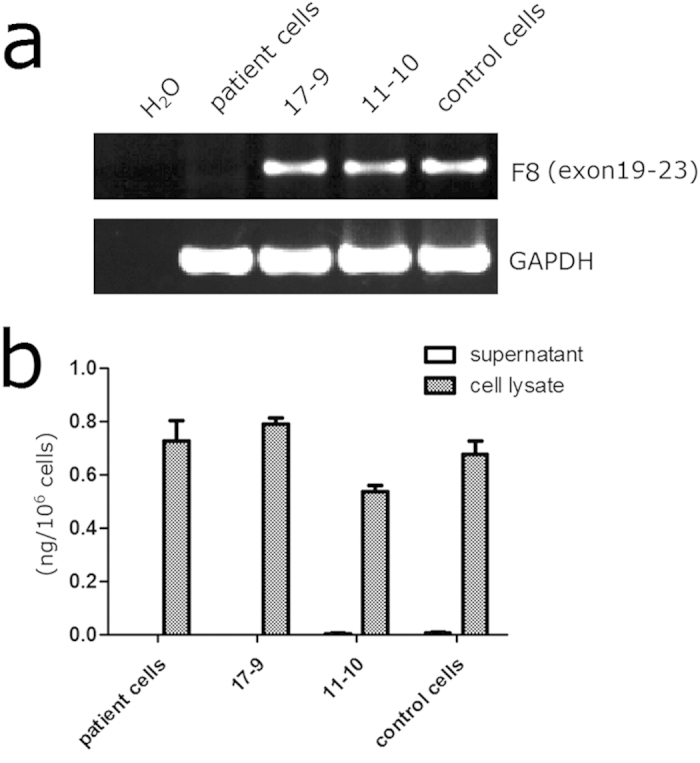
*F8* expression in iPSCs. (**a**) RT-PCR analysis of transcripts across the junction of exon 22 and intron 22 of *F8*. The primers were based on exon 19 and exon 23 sequences. The transcripts were undetectable in the initial patient-specific iPSCs, but could be detected in gene corrected clones as well as non-hemophiliac control iPSCs. Agarose gel results were cropped from the same gel and the full-length gel was presented in Supplementary Fig. S7e. (**b**) ELISA of FVIII related proteins in cell lysates and culture supernatants of the iPSCs. The proteins could only be detected in cell lysates of the iPSCs, 17-9 and 11-10 are gene corrected clones without PGK-Neo cassette.

The transcripts crossing the exon 22–23 boundary could not be detected in patient-specific iPSCs, indicating a typical transcription pattern of *F8* with Inv22. However, these boundary-crossing transcripts were detected in the gene-corrected clones as well as normal control cells (Fig. 5a). We collected the cell lysates and culture supernatants and tested the protein production using ELISA. The target signals were detected in cell lysates of the patient, gene-corrected and control iPSCs, while the secretory FVIII-related proteins could not be demonstrated in the culture supernatants of all these cells (Fig. 5b). However, a recent paper described a detectable FVIII secretion in normal iPSCs^14^. The discrepancy might be caused by different cell lines and detection methods being used.

**Figure 6:**
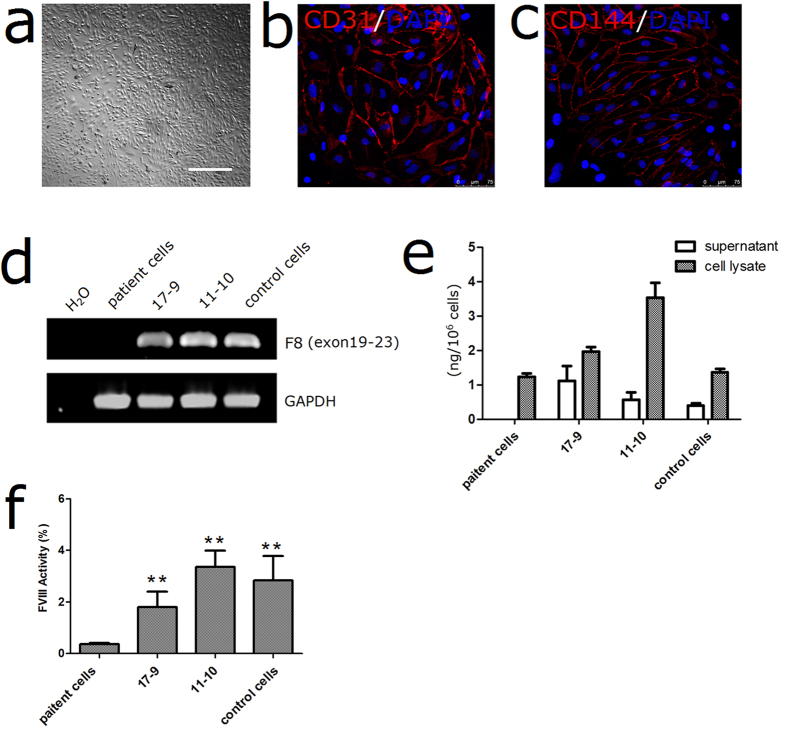
Differentiation of the iPSCs into ECs. (**a**) Morphology of the ECs after magnetic-activated cell sorting. Scale bar represents 200 μm. (**b**,**c**) Immunostaining of ECs expressing markers for CD31, CD144, DAPI was used to visualize the nucleus. (**d**) RT-PCR analysis of *F8* expression. Agarose gel results were cropped from the same gel and the full-length gel were presented in Supplementary Fig. S7f. (**e**) ELISA to measure FVIII antigen. The secretion of FVIII proteins was restored in gene corrected clones. (**f**) FVIII activity of the supernatant from the differentiated ECs. (**p < 0.01).

**Figure 7:**
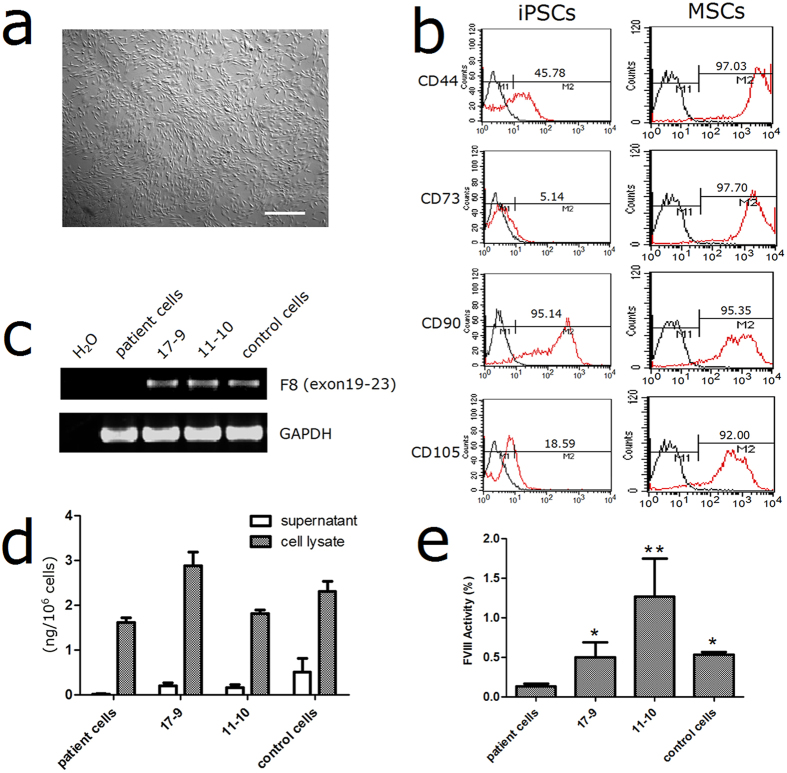
Differentiation of the iPSCs into MSCs. (**a**) Morphology of the MSCs derived from iPSCs. Scale bar represents 200 μm. (**b**) Flow cytometric analysis of immunological surface markers used to define human MSCs, both iPSCs and iPSCs-derived MSCs were analyzed. (**c**) RT-PCR analysis of *F8* expression. Agarose gel results were cropped from the same gel and the full-length gel was presented in Supplementary Fig. S7g. (**d**) ELISA to measure FVIII antigen. The MSCs derived from the corrected clones and non-hemophiliac control iPSCs can release FVIII protein into the supernatant. (**e**) FVIII activity of the concentrated supernatant from the differentiated cells. (*p < 0.05; **p < 0.01).

We differentiated both the patient original and gene-corrected iPSCs into ECs to test the *F8* expression^15^. The ECs were sorted using CD31 microbeads and characterized by immunostaining of CD31 and CD144 (Fig. 6a–c). RT-PCR revealed that the *F8* transcription was rescued in ECs derived from the gene-corrected iPSCs (Fig. 6d). Although FVIII-related proteins could be synthesized in the ECs derived from the original iPSCs, they could not be released into the supernatants. However, the ECs from the gene-corrected iPSC clones 17-9 and 11-10 secreted the FVIII protein at levels of 1.12 ± 0.44 ng/10^6^ cells and 0.57 ± 0.22 ng/10^6^ cells respectively (Fig. 6e). Recent researches reported that the patients with intron 22-inverted *F8* express the entire primary amino acid sequence of FVIII as two non-secreted polypeptide chains^16^,^17^. Our results show that the gene correction not only corrected the transcription of inverted *F8*, but also restored the secretion of FVIII protein. Furthermore, we assayed the FVIII activity with the culture supernatant, and found that functional protein could be secreted by ECs (Fig. 6f). Besides ECs, human MSCs had been proven to contribute to the functional FVIII pool^18^. As MSCs are multipotent cells and have the ability to proliferate extensively, they are considered to be used as a vehicle for HA gene therapy^19^. We differentiated the iPSCs into MSCs as previously described (Fig. 7a)^20^. The iPSC-derived MSCs expressed high level of the typical MSC markers such as CD44 (97.03%), CD73 (97.70%), CD90 (95.35%) and CD105 (92.00%) after differentiation (Fig. 7b; Supplementary Fig. S6). The MSCs derived from the gene-corrected iPSC clones showed a normal transcription pattern and secreted the FVIII proteins at 0.20 ± 0.06 ng/10^6^ cells and 0.16 ± 0.08 ng/10^6^ cells (Fig. 7c,d). And coagulation activities of the FVIII proteins in the supernatants from the gene corrected MSCs were detectable by FVIII activity assay (Fig. 7e).

## Discussion

As one of the most attractive models for gene therapy research, hemophilia has been studied extensively based on different vectors and different strategies. While AAV-mediated approaches in HB are extremely encouraging, there is no similar data for factor VIII gene therapy. Reasons for this include difficulties of packaging the large FVIII cDNA, achieving adequate transgene expression and preventing the frequent anti-FVIII immunity. In previous researches, therapeutic levels of FVIII have been obtained by intravenous administration of adenoviral vectors in both mice and dogs^21^,^22^,^23^. Lentiviral-based gene transfer strategies are attractive too, especially using the platelet as a target as previously reported^24^. Although viral-mediated approaches have led the way in hemophilia gene therapy, there are still advantages of using an alternative delivery vehicle including the fact that *ex vivo* host cell transduction will avoid the immune responses to the vector.

Severe HA, accounting for about 60% of the HA population, is a disabling and even lethal disease without a cure, making it necessary to develop gene-based therapeutic approaches. Interestingly, although more than 2800 different mutations of *F8* have been identified, nearly half of the severe HA cases were caused by Inv22 (Human Gene Mutation Database. – http://www.hgmd.cf.ac.uk/ac/gene.php?gene=F8.). Individuals with Inv22 synthesize FVIII-related proteins as two polypeptides intracellularly but do not secret them at all^16^,^17^. In the present study, we developed an approach to correct the Inv22 mutation by integrating the last four exons at the exon22-intron22 junction, aiming at almost half of the patients with severe HA.

Kim and colleagues described a direct reversion of the intron 1 inversion of *F8*, which accounts for 1–4% of severe HA cases^25^. They firstly modeled a 140-kb intron 1 inversion and then reverted it in the normal iPSCs using the TALENs targeting the intron 1 homolog responsible for this type of inversion. The innovative reversion is proved to be a gene therapy process as it rescued the expression of *F8*. However, without using a resistant gene, only 1.3% (4 in 300 clones) targeting efficiency was achieved in the reversion experiment. Moreover, the TALEN sites remain intact after gene manipulation, raising the possibility that any residual activity of the TALENs will cleave the target site again and lead to a second round of inversion or indels. In their study indeed, indels were found in two of the four reverted clones. Inv22 might be reverted in a similar way, however, a much larger inverted fragment and three copies of the responsible homolog will lead to even lower efficiency and other unexpected consequences. By comparison, using our described homology directed repair strategy, a high targeting efficiency of over 50% is achieved and the PGK-Neo cassette can be removed from the genome efficiently.

Human iPSCs can be expanded unlimitedly in culture and hold the ability to differentiate into all somatic cells^26^. Patient-specific iPSC is a perfect model for testing such a gene-correction approach described above. Protocols of gene targeting in pluripotent stem cells have established with or without artificial nucleases^27^,^28^. There are three types of artificial nucleases available now, zinc finger nucleases (ZFNs), TALENs and clustered regularly interspaced short palindromic repeats (CRISPR)/Cas-based RNA-guided DNA endonucleases^29^,^30^,^31^. ZFNs are the first chimeric nuclease that played an important role in targeted gene manipulation over the past two decades^32^. However, the low efficiency and laborious selection process makes it only available at Sangamo BioSciences and several professional laboratories^33^. TALENs are much easier to design and proved to be significantly more mutagenic than ZFNs^34^. In the present study, we used TALENs to stimulate HR and achieved high targeting efficiencies of more than 50%. Although off-target activity of L1R1 was detected in HEK293T cells, we can still select the single iPSC clones without modification at these sites after sequencing.

Unlike other coagulation factors, human FVIII is not synthesized in hepatocytes. ECs are the major contributor of FVIII in liver, lung and other tissues^35^,^36^,^37^. A previous research showed that expression of FVIII in the vascular endothelium restored normal levels of FVIII in plasma of FVIII deficient mice^38^. Furthermore, von Willebrand factor (vWF) synthesized by ECs serves as a carrier protein to stabilize FVIII^39^. We therefore differentiated the iPSCs into ECs and found that the FVIII-related proteins could be synthesized intracellularly but not be secreted into the culture supernatant of the uncorrected iPSC-derived ECs. By contrast, the gene-corrected parallel cells, as well as the normal control cells, could synthesize and secrete FVIII protein efficiently. Although previous studies in conditional knockout mice indicated that ECs are possibly the exclusive source of plasma FVIII in mice^40^,^41^,^42^, secretion of FVIII in human could be detected in MSCs and peripheral blood mononuclear cells^16^,^18^. In our study, both the gene-corrected and control MSCs were able to secrete FVIII. We found that the FVIII production is obviously different in the differentiated cells derived from different corrected iPSC clones. It has been indicated that the FVIII production capacities are different among ECs purified from different vascular beds in human. For instance, the majority of FVIII is produced and secreted by hepatic sinusoidal endothelial cells, while the FVIII production in umbilical vein endothelial cells is undetectable^36^. Similar discrepancy was observed by quantify FVIII level in MSCs derived from different tissues^18^. Methods for *in vitro* directed differentiation of the tissue-specific ECs or MSCs are not available now. We therefore reason that although the ECs and MSCs were characterized by classic markers, the constitution of the EC or MSC population derived from different clones may be different.

Our results also showed that FVIII could be synthesized but not secreted in iPSCs, of which the underlying mechanism is still largely unknown. The secreted FVIII of the gene-corrected ECs or MSCs is comparable to that of the normal control cells, though is significantly lower than that of the viral transduced cells. Viral vectors often transfer multi-copy transgene with a strong promoter into the target cells, of which the FVIII expression should be much higher than those of the normal control or gene-corrected cells with only one copy of *F8* under the endogenous promoter. Large amount of cells will be needed to achieve therapeutic level of FVIII based on the secretion data in our *in vitro* experiments. However, it is well acknowledged that the cell performance *in vivo* is quite different from *in vitro*, even though has not been evidenced in our study. Some researches showed that around 10% of circulating FVIII was achieved *via* transplantation of ECs and/or MSCs. Xu *et al*. injected the normal iPSC-derived endothelial cells into HA mice, plasma FVIII levels increased up to 8%–12% of wild type^43^. In an experiment performed in HA sheep, transplanted MSCs homed to the sites of ongoing injury/inflammation to release FVIII, promoting the improvement of hemarthrotic joints^44^. Follenzi *et al*. showed that bone marrow transplantation corrected HA through donor-derived mononuclear cells and MSCs^45^.

In summary, we performed a TALEN-mediated correction of Inv22 by targeting a complemented sequence at the junction of exon 22 and intron 22 with a high efficiency in HA patient-specific iPSCs. The gene-corrected iPSCs retained a normal karyotype following removal of drug selection cassette using a Cre-LoxP system. And both *F8* transcription and FVIII secretion were rescued in the ECs and MSCs derived from the gene-corrected iPSCs. This is the first report of an efficient *in situ* genetic correction of large inversion mutation using a strategy of targeted gene addition, while further experiments need to verify the safety and effectiveness. And hopefully, some non-viral and integration-free approaches for reprogramming of urine cells by plasmid transfection are available now^46^, facilitating our approach’s potential of being used clinically.

## Methods

### Generation of patient-specific iPSCs

Peripheral blood was collected from severe HA patients following informed consent. Genomic DNA was extracted for screening of Inv22 by inverse shifting-polymerase chain reaction (IS-PCR) as previously reported^8^. Sterile urine was collected from a 51-year-old male diagnosed as distal Inv22. Urine cells were pelleted and seeded on gelatin coated 12-well plate using medium described previously^9^. The outgrowth epithelial cells were used for iPSC generation between passages 1-3. For viral package, HEK293T cells of 80% confluence were transfected with pMXs vectors (pMXs-hSox2, pMXs-hOCT4, pMXs-hKlf4, pMXs-hc-Myc and pCL-Eco, all from Addgene) using lipofectamine 2000 (Life Technologies). Urine cells were seeded at a density of 6,000/cm^2^ in REGM BulletKit (Lonza) on 6-well plate on the day before infection. Viral supernatants were collected 48 and 72 hours after transfection and used for infection of urine cells in the presence of 8 μg/ml polybrene (Sigma-Aldrich). Four days after the initial infection, cells were trypsinized and 10,000 cells were seeded in 10 cm dish containing feeders and ES medium. Two weeks after replating, ESC-like clones were mechanically picked up and expanded for further characterization. All experimental protocols were approved by the Ethics Committee of the state key laboratory of medical genetics of China, and all participants provided written informed consent to participate in this study. The methods were carried out in accordance with the approved guidelines.

### Characterization of patient-specific iPSCs

For immunofluorescent staining, cells on glass slides were fixed in 4% paraformaldehyde for 20 minutes and permeabilized with 0.1% Triton-X 100 for 15 minutes. After blocking in 5% bovine serum albumin for 30 minutes, the cells were incubated with the first antibody at room temperature for 1 hour. After appropriate wash, samples were treated with secondary antibodies for 1 hour. DNA was visualized using 4, 6-Diamidino-2-phenylindole (DAPI) and cells were mounted under coverslips using Fluoromount-G (SouthernBiotech). For teratoma formation, cells on 10 cm dish were dissociated using 0.05% Trypsin/EDTA (Life Technologies). Pelleted cells were resuspended in 140 μL DMEM/F12 and 70 μL Matrigel^TM^ and injected subcutaneously into the hind legs of immunocompromised mice. Two months later, the formed teratomas were harvested and fixed in 4% paraformaldehyde. Then the tissues were sectioned and analyzed after stained with hematoxylin and eosin. All procedures regarding the care and use of animals are in accordance with institutional guidelines. This study was approved by the Ethics Committee of State Key Laboratory of Medical Genetics of China.

### TALEN design and assembly

Two pairs of TALENs targeting the exon22-intron22 junction of *F8* were designed using ZiFiT software (http://zifit.partners.org/ZiFiT/) and constructed using REAL Assembly TALEN Kit from Joung lab^11^. For gene disruption assay, HEK293T cells were transfected with TALENs using Lipofectmaine 2000. Genomic DNA was extracted 36 hours after transfection. The genomic region encompassing the junction was PCR amplified and cloned. Thirty colonies for each transfection were picked up and sequenced after transformation. Alternatively, the cleavage activity could also be evaluated by T7 endonuclease I (T7E1) assay. Briefly, the genomic region encompassing the nuclease target sites was PCR amplified. Then the purified DNA fragments were digested with the mismatch-sensitive T7E1 after a programmed denaturing-annealing cycle. After electrophoresis, both the cleaved and uncleaved fragments were quantified using a gel imaging system. The TALEN activity was estimated as previously described^47^.

### Construction of a donor vector for gene correction

An 806 bp left homology arm and a 909 bp right arm with an NheI site were amplified from human genomic DNA. The coding sequence of exon 23–26 plus an SV40 polyA signal was amplified from the vector hFVIII-BDDAK39 we constructed previously^48^. Then the three fragments were ligated through an overlap-PCR. An NheI flanked PGK-Neo cassette was constructed by an overlap-PCR after amplification of PGK and Neo from pSicoR pGK puro (addgene) and hFVIII-BDDAK39 respectively. Then the PGK-Neo cassette was inserted into NheI linearized donor vector.

### Gene targeting and excision of PGK-Neo

Patient-specific iPSCs on Matrigel^TM^ coated dishes were used for transfection. After incubation with 10 μM Y27632 (Sigma-Aldrich) for 2 hours, cells were detached with trypLE^TM^ Select (Life Technologies) and counted. Then the cells were resuspended with 100 μL Human Stem Cell Nucleofector Kit 2 (Lonza) and nucleofected using Nucleofector II (Lonza) set at program B016. For each experiment, 5 μg of linearized donor vector was used with or without the 5 μg of the TALENs. The transfected cells were plated on PMEF-NL (Millipore) feeders in ES medium containing 10 μM Y27632. G418 selection (50 μg/ml, Calbiochem) was started 48–72 hours after transfection. About two weeks later, resistant clones were picked and expanded for further analysis.

For excision of PGK-Neo, cells on matrigel were detached with trypLE^TM^ Select and pelleted. Then the cells were resuspended with 100 μL Human Stem Cell Nucleofector Kit 2 with 5 μg pCAG-cre (addgene) and nucleofected using Nucleofector II set at program B016. Ten thousand of nucleofected cells were seeded on 6 cm dish with feeders in ES medium containing 10 μM Y27632. After two weeks of continuous culture without drug selection, single clones were picked and expanded for further analysis.

### PCR and Southern blot detection of gene correction

Genomic DNA was isolated from the cells on 24-well plates using phenol/chloroform extraction. PCR was performed using LA Taq DNA polymerase (TaKaRa) according to the manufacturer’s recommendations. Two pairs of primers were used to detect the first step of the targeted gene correction. For wild type iPSCs, no product could be amplified using either of the primer pairs. After homologous recombination (HR), a 1.3 kb and a 1.4 kb fragment could be detected using primers F1/R1 and F2/R2 respectively. Primers F3 and R3 were used to detect the cre-excision of PGK-Neo cassette. The PCR product for clones underwent the first step correction will be 2.5 kb. If the Neo cassette is successfully removed, the expected size of the PCR product will be only 0.53 kb.

For Southern blot analysis, 5 μg genomic DNA digested with AflII restriction enzyme (New England Biolabs) was electrophoresed on a 0.8% agarose gel overnight. Then the samples were capillary transferred to positively charged nylon membranes (Roche Diagnostics). The blots were hybridized with DIG-dUTP labeled probes corresponding to the 3′ homologous arm overnight at 42 °C. After incubation with AP-conjugated DIG-Antibody (Roche Diagnostics) and appropriate washing, the signals were detected using CDP-Star (Roche Diagnostics) as a substrate for chemiluminescence. The expected fragment sizes are 1.8 kb, 4.6 kb and 2.7 kb for patient cells, corrected clones with PGK-Neo and corrected clones after the removal of PGK-Neo cassette respectively.

### Derivation of ECs and MSCs from iPSCs

The differentiation of the iPSCs into ECs was carried out as previously described with minor modifications^15^. Briefly, iPSCs were detached with dispase and transferred onto 5 days old OP9 cells in α-MEM supplemented with 10% FBS and 100 μM monothioglycerol (Sigma-Aldrich). The medium was refreshed on every other day. On day 9, the differentiated cells were harvested by treatment with 0.05% trypsin/EDTA for 15 minutes at 37 °C. After being filtered through a 40 μm cell strainer (BD Biosciences), the cells were subjected to magnetic-activated cell sorting using CD31 MicroBead kit (Miltenyi Biotec) according to the manufacturer’s instructions. The sorted cells were seeded on fibronectin coated plates in Human Endothelial-SFM supplemented with 20 ng/ml bFGF, 10 ng/ml EFG and 10 μg/ml human plasma fibronectin (all from Life Technologies).

To differentiate iPSCs into MSCs, cells were trypsinized and reseeded on gelatin coated dishes in KnockOut DMEM supplemented with 10% knockout serum replacement, 1% nonessential amino acids, 1% penicillin/streptomycin, 2 mM L-glutamine, 0.1 mM β-mercaptoethanol, 20 ng/ml bFGF and 20 ng/ml EFG (all from Life Technologies). After being passaged 4–5 times with 0.05% Trypsin/EDTA, cells were sent for flow cytometry analysis with a FACSCalibur flow cytometer (BD Biosciences)^20^.

### RT-PCR

Total RNA was extracted using Trizol reagent (Sigma-Aldrich) and reverse transcribed using the primers 5′-GGGGGTGAATTCGAAGGTAG-3′ and Oligo(dT)_15_. Primers based on exon 19 and exon 23 sequences were used to detect the transcripts across the exon 22–23 boundary.

### FVIII assay of culture supernatant and cell lysate

Twenty-four hours old culture supernatants were collected from 12-well plates. Then the cells were trypsinized and counted. After washed with PBS, the pelleted cells were resuspended in 500 μL sample dilunent for ELISA (CEDARLANE) and lysed by 3 freeze-thaw cycles. All samples were collected in triplicate. ELISA was performed using Paired Antibodies for ELISA-Factor VIII:C (Cedarlane Laboratories) according to the manufacturer’s instructions. Reference curves were constructed using serial dilutions of normal pooled plasma, with correlation coefficient (R^2^) of at least 0.980 using a semi-log fit. For FVIII activity assay, cell-free supernatant was collected twenty-four hours after medium change and concentrated 5 times using a centrifugal filter (Millipore). Coagulation Factor VIII Deficient Plasma (Siemens) and Destiny Max™ Haemostasis Analyser (Tcoag) were used to examine the activated partial thrombo-plastin time (aPTT), for calculating the FVIII activity according to the manufacturer’s instructions.

## Additional Information

**How to cite this article**: Wu, Y. *et al*. *In situ* genetic correction of *F8* intron 22 inversion in hemophilia A patient-specific iPSCs. *Sci. Rep.* **6**, 18865; doi: 10.1038/srep18865 (2016).

## Supplementary Material

Supplementary Information

## Figures and Tables

**Table 1:** Summary of the three experiments of targeted genetic correction.

TALENs	Cells transfected	Total clones	Clones analysed	Corrected clones	Targeting efficiency
L1R1	6 × 10^6^	568	16	10	62.5%
L2R2	5 × 10^6^	55	17	9	52.9%
−/−	5 × 10^6^	30	30	0	0
