# Supplementary material for: In situ genetic correction of F8 intron 22 inversion in hemophilia A patient-specific iPSCs
Source: Sci Rep. 2016 Jan 8;6:18865. doi: 10.1038/srep18865 (PMC4705535; doi:10.1038/srep18865)
Supplement: Supplementary Information [file srep18865-s1.pdf]

Supplementary Information

In situ genetic correction of *F8* intron 22 inversion in hemophilia A patient-specific iPSCs

Yong Wu[1,2], Zhiqing Hu[1], Zhuo Li[1], Jialun Pang[1], Mai Feng[1], Xuyun Hu[1], Xiaolin Wang[1], Siyuan Lin-Peng[3], Bo Liu[1], Fangping Chen[2], Lingqian Wu[1,3,*] & Desheng Liang[1,*]

[1]State Key Laboratory of Medical Genetics, School of Life Sciences, Central South University, Changsha, Hunan, China

[2]Department of Hematology, Xiangya Hospital, Central South University, Changsha, Hunan, China

[3]Hunan Jiahui Genetics Hospital, Changsha, Hunan, China

Correspondence and requests for materials should be addressed to Desheng Liang or Lingqian Wu, State Key Laboratory of Medical Genetics, Central South University, 110 Xiangya Road, Changsha, Hunan 410078, China. Telephone: +86-731-84805252; Fax: +86-731-84478152; e-mail: liangdesheng@sklmg.edu.cn or wulingqian@sklmg.edu.cn

Supplementary Figures

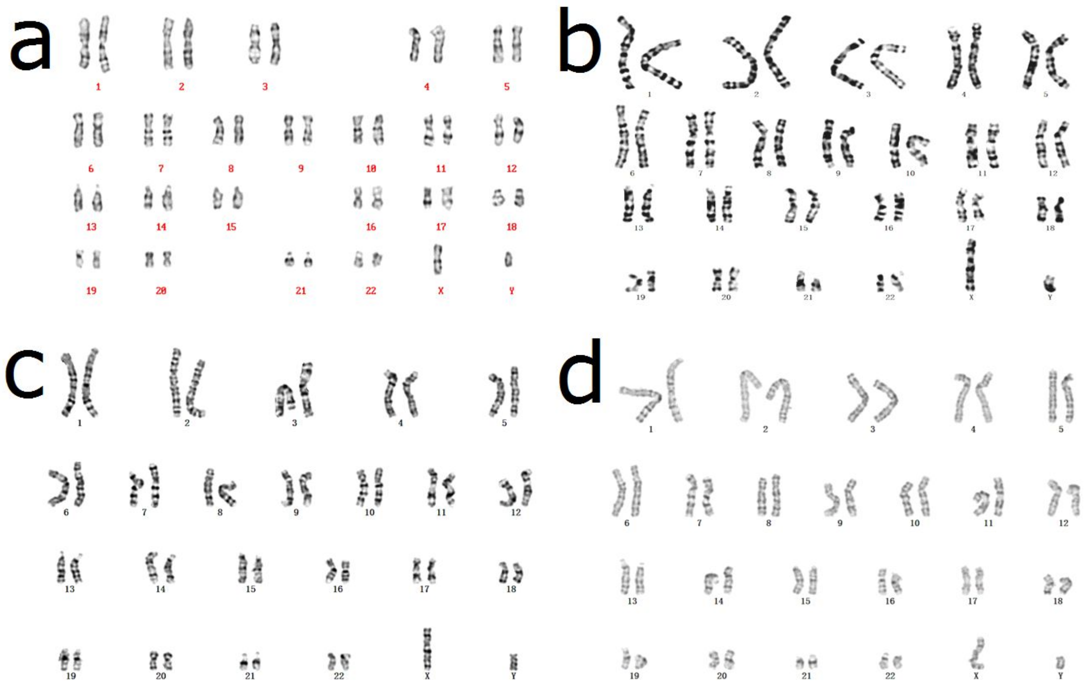

Supplementary Figure S1. Karyotyping of the iPSCs

(**a**) Karyotype of the initial patient-specific iPSCs. (**b**) IPSCs after the first step of gene targeting. (**c**-**d**) Gene corrected clones after the removal of PGK-Neo cassette. No robust translocation was found in all the karyotypes.

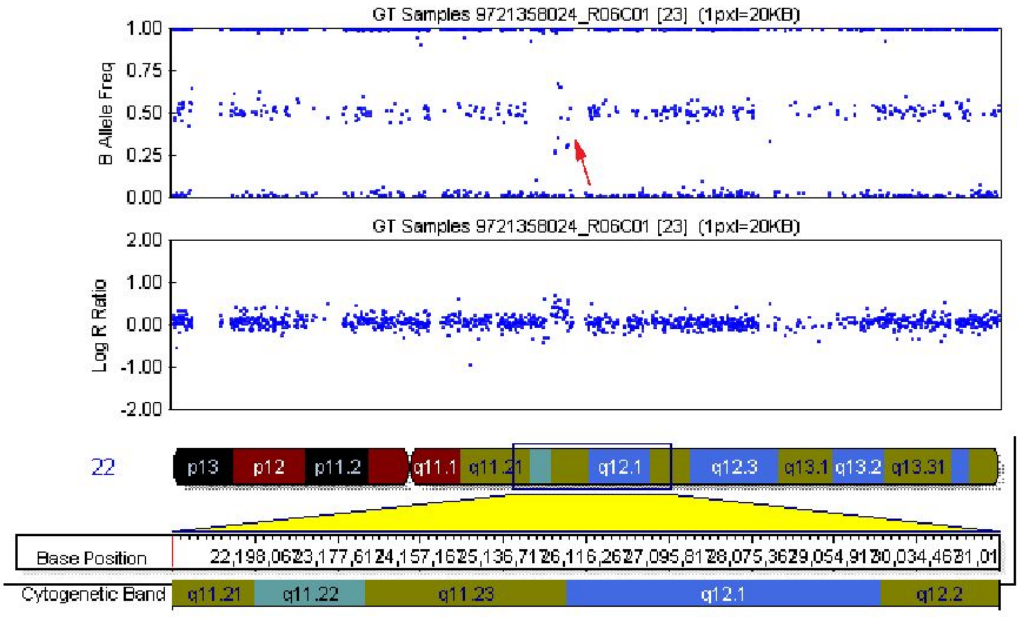

Supplementary Figure S2. Copy number variation (CNV) analysis using Illumina Human OmniZhongHua-8 Beadchip

The patient urine cells, reprogrammed iPSCs, two genetic corrected clones and two clones after Neo excision were analyzed. B Allele Frequency and Log R show a gain of 295,170 bp (nt. 25615710-25910879) in 22q11.23 (Red arrow) in all the samples including the initial urine cells. No further CNV was detected after reprogramming, genetic correction or Neo deletion.

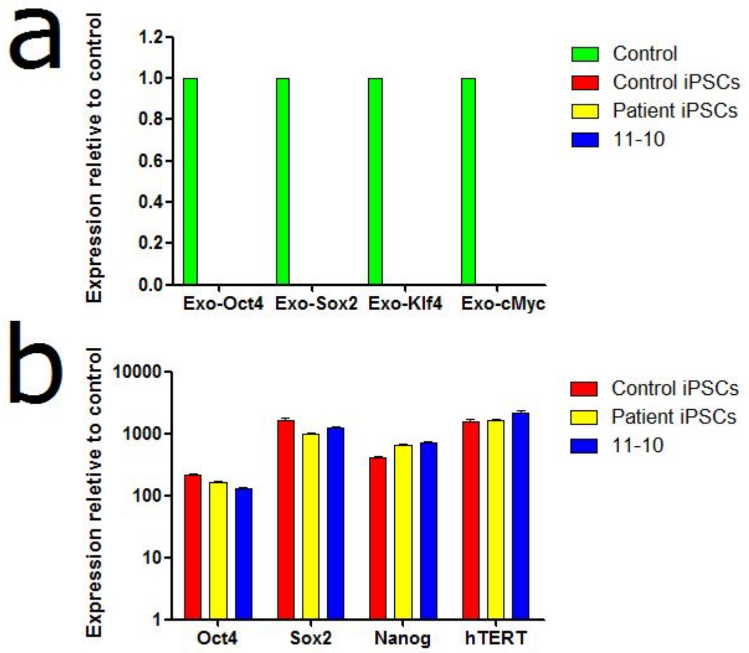

Supplementary Figure S3. Quantitative real-time PCR (qRT-PCR) for pluripotent genes.

(**a**) qRT-PCR showing silencing of the exogenous transgenes in iPSCs. Values are referred to transduced cells extracted at day 4. (**b**) qRT-PCR for endogenous pluripotent genes in iPSCs. Values are referred to urine cells. A hiPSC line purchased from ATCC was used as a control (http://www.atcc.org/products/all/ACS-1011.aspx).

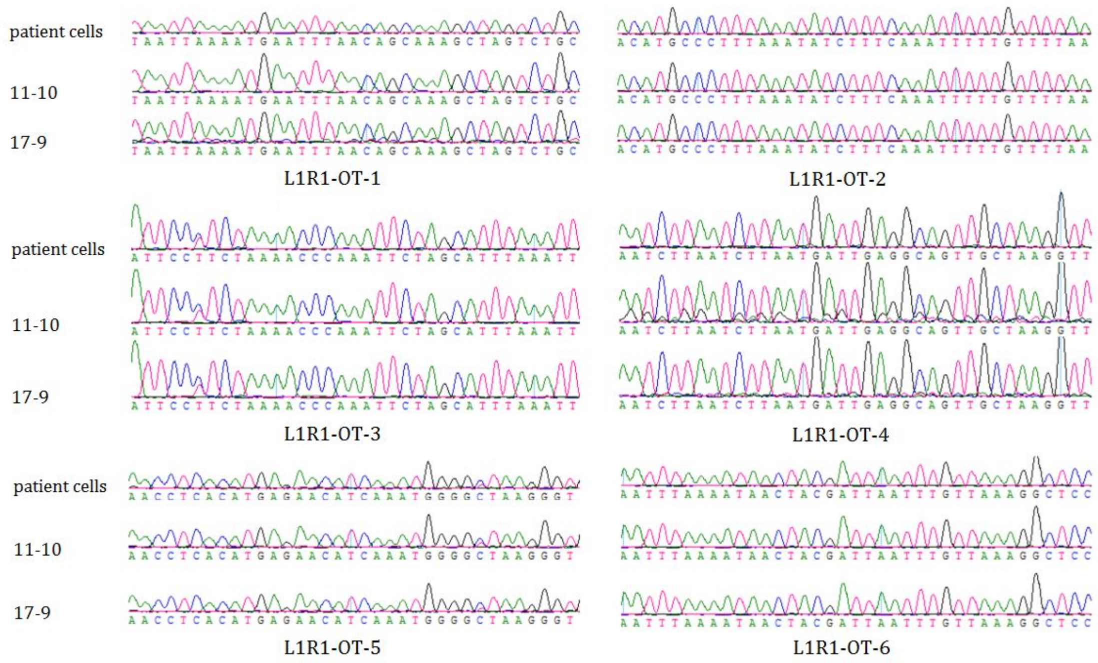

Supplementary Figure S4. Sanger sequencing of potential off-target sites after genetic correction using L1R1

The genomic region encompassing the junction was PCR amplified and sequenced. Both the initial patient cells and the clones after TALEN-stimulated genetic correction were analyzed. No indels at the sites were induced in the two clones.

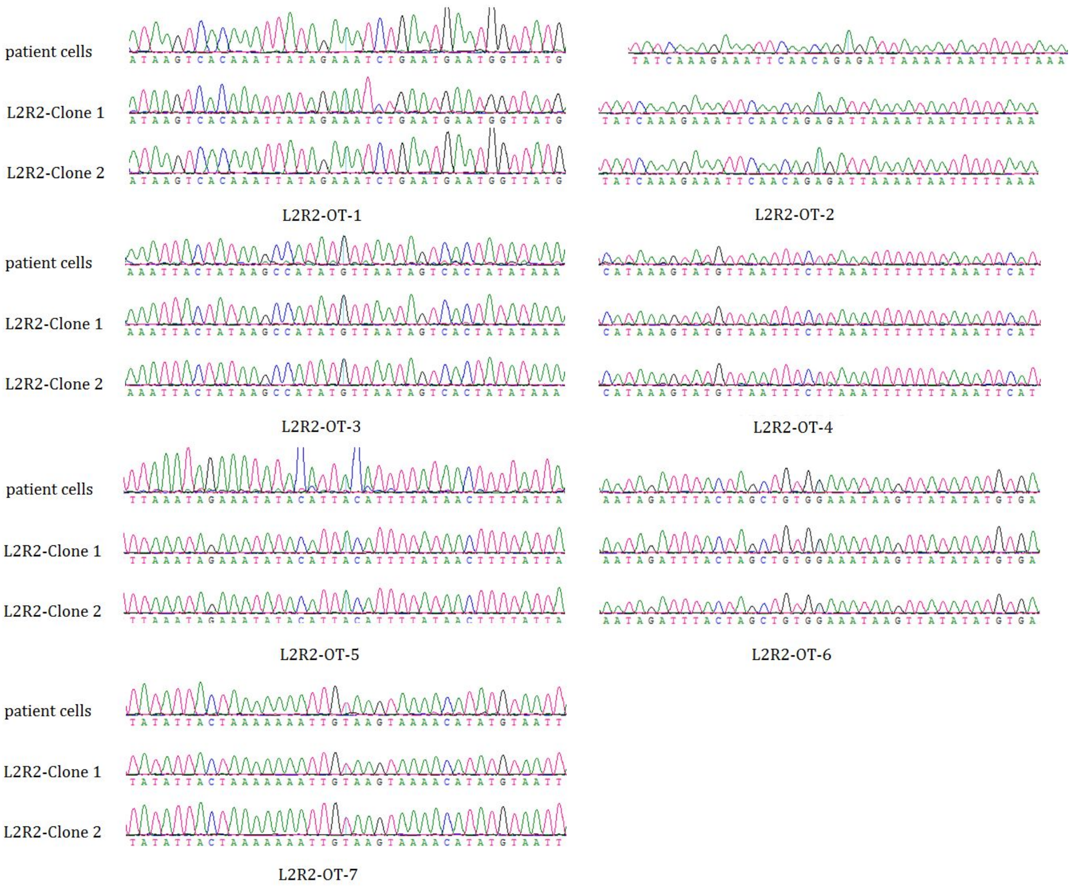

Supplementary Figure S5. Sanger sequencing of potential off-target sites after genetic correction using L2R2

The genomic region encompassing the junction was PCR amplified and sequenced. Both the initial patient cells and the clones after TALEN-stimulated genetic correction were analyzed. No indels at the sites were induced in the two clones.

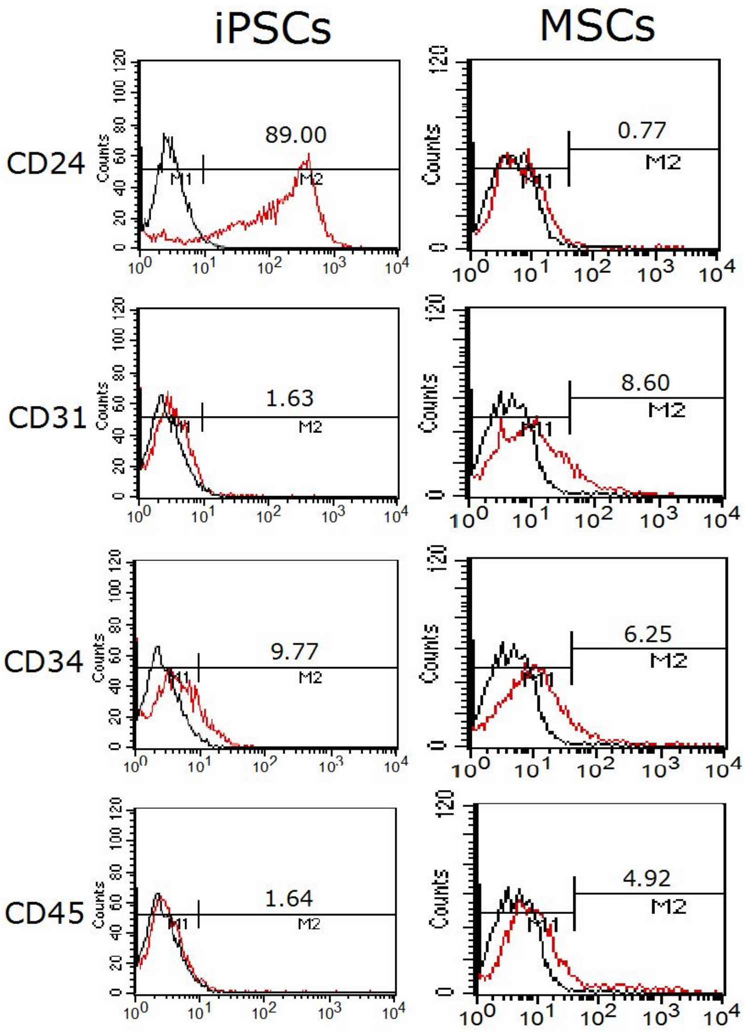

Supplementary Figure S6. Flow cytometry analysis of iPSCs and MSCs

Flow cytometry analysis of early stem cell marker CD24, EC expressing marker CD31, and hemopoietic stem cell markers CD34 and CD45 on both iPSCs and MSCs.

a

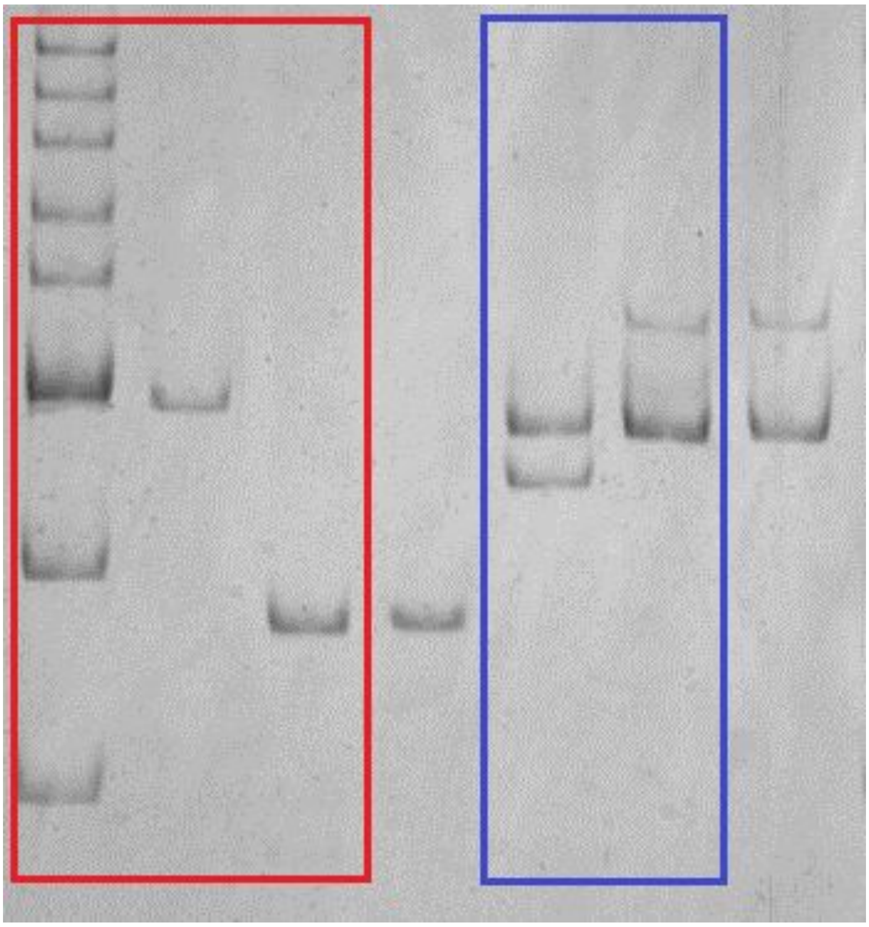

b

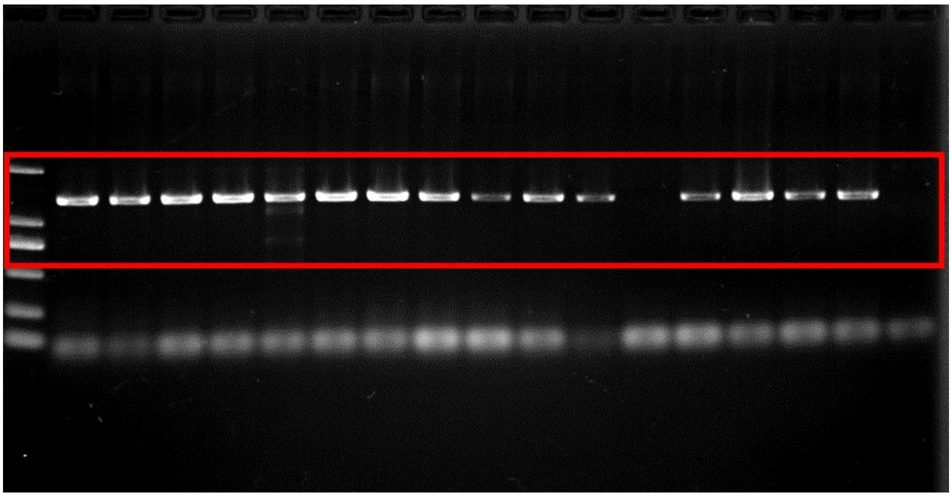

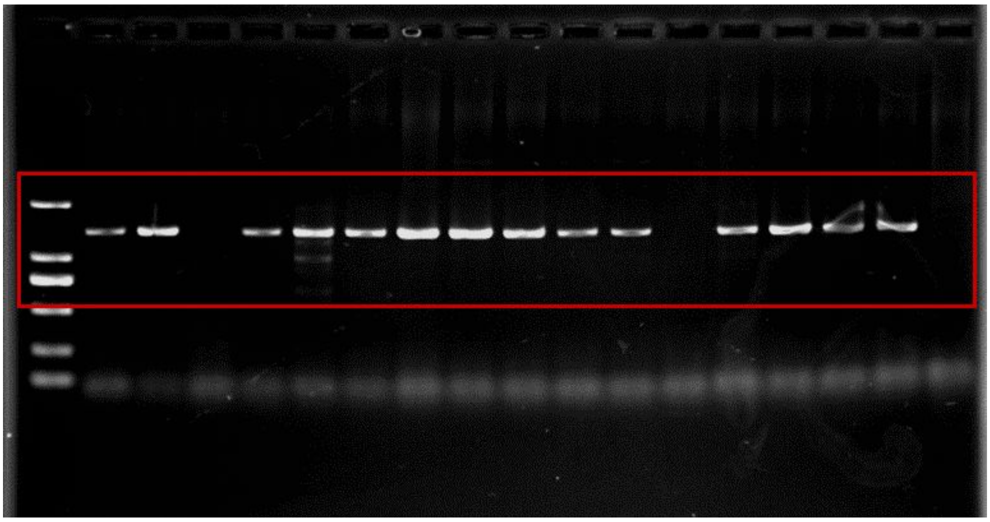

c

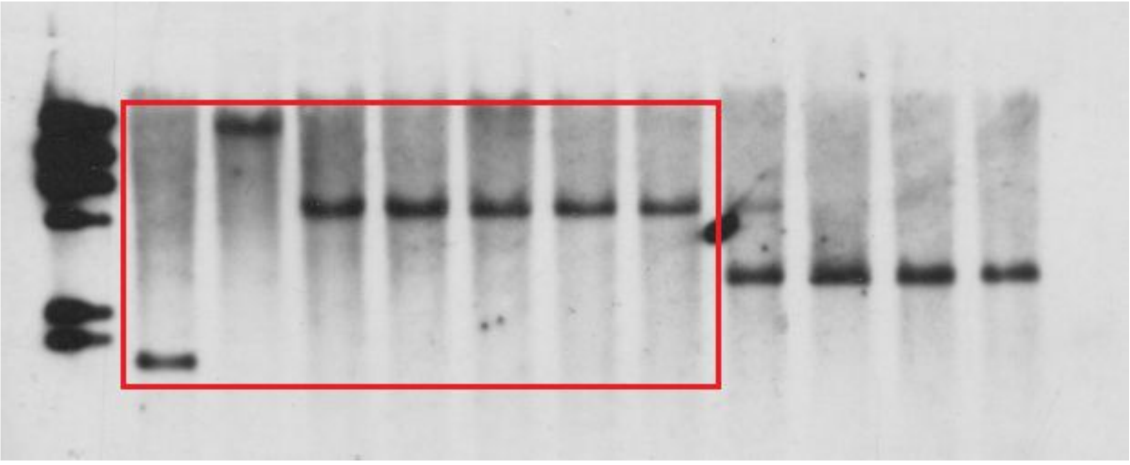

d

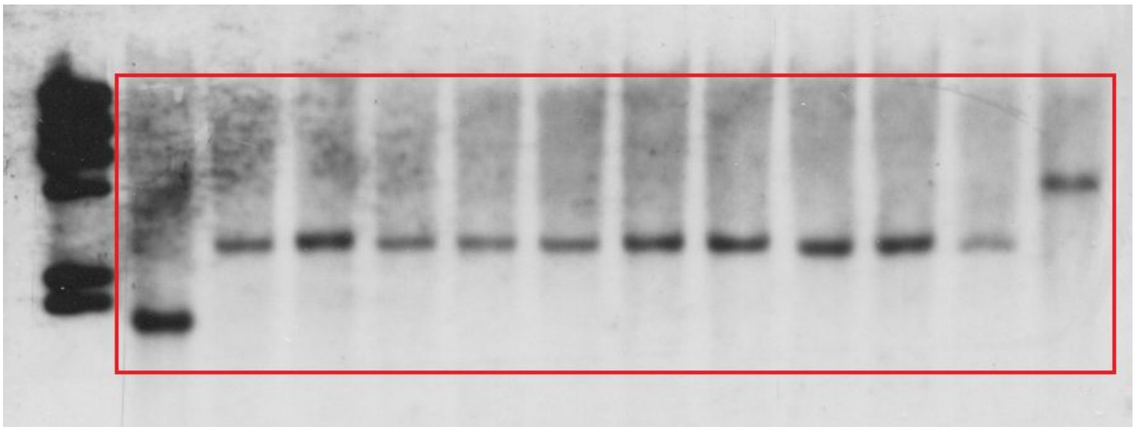

e

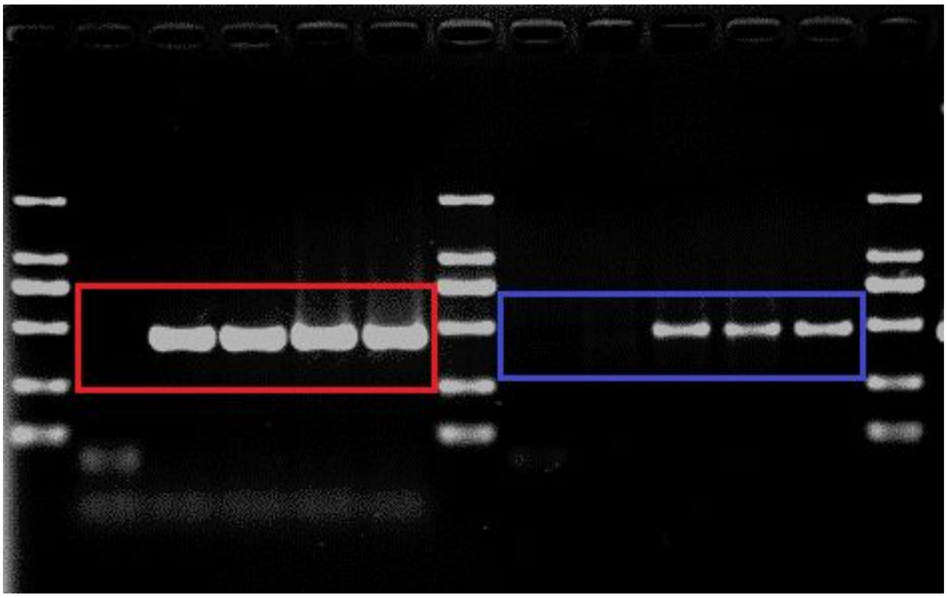

f

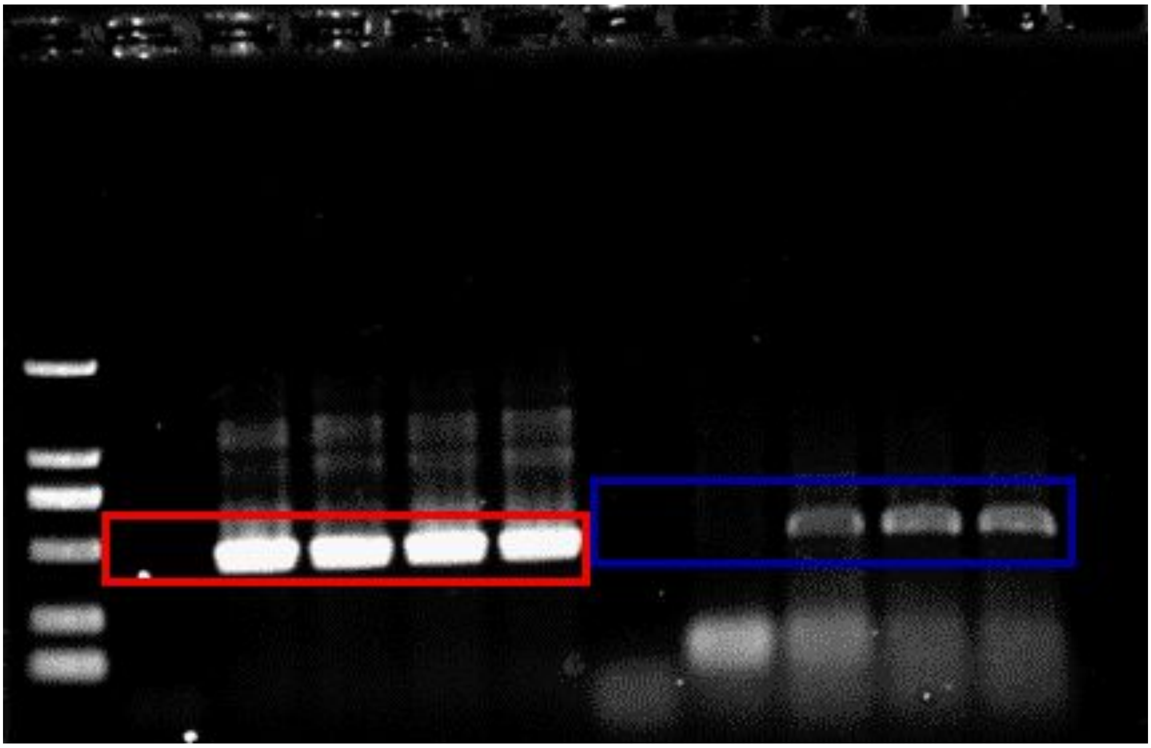

g

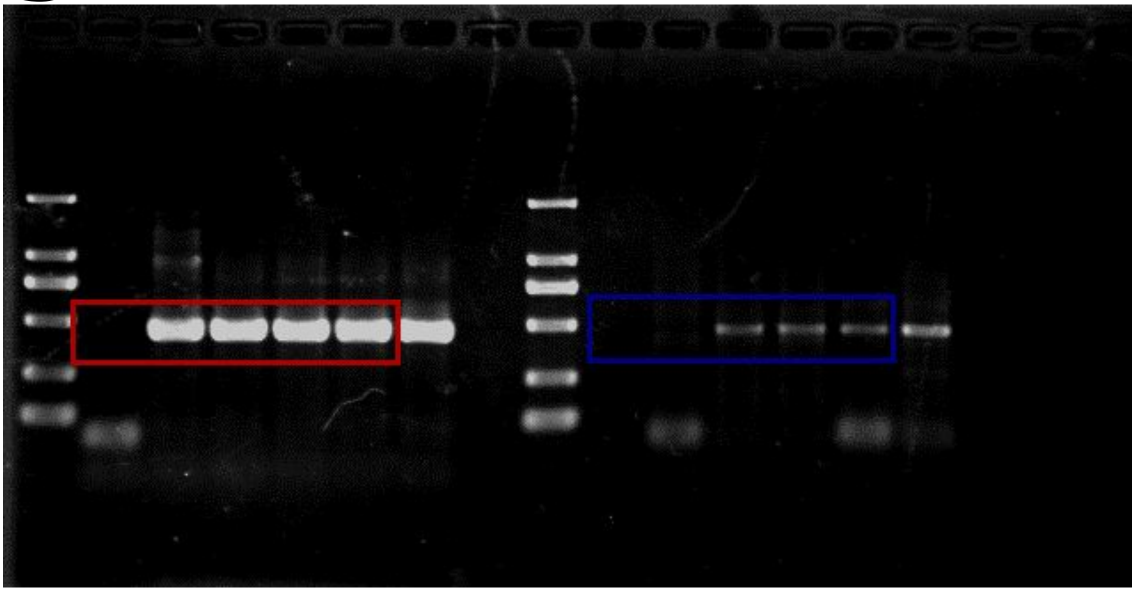

Supplementary Figure S7. Full-length gels and blots which have been cropped in the main text are presented.

(**a**) IS-PCR results in **Fig. 1a** of the main text, red lines (Inv22 test) and blue lines (Complementary test) represent cropping lines. Sample of another previously diagnosed Inv22 patient was loaded as a control (out of the lines), which was not used in the main figure. (**b**) **Fig. 4a** in the main text, PCRs using F1R1 (upper pannel) and F2R2 (down pannel) were indicated by red lines. All the gels have been run under the same experimental conditions. (**c**) **Fig. 4c** in the main text was shown here. (**d**) Southern blotting result of **Fig. 4f.** (**e**) RT-PCR results in **Fig. 5a** in the main text. GAPDH (453 bp) and F8 (cross exon-22-23 junction, 478 bp) were indicated using red and blue lines respectively. (**f**) RT-PCR results of ECs in **Fig. 6d** in the main text. GAPDH (453 bp) and F8 (cross exon-22-23 junction, 478 bp) were indicated using red and blue lines respectively. (**g**) RT-PCR results of MSCs in **Fig. 7c** in the main

text. GAPDH (453 bp) and F8 (cross exon-22-23 junction, 478 bp) were indicated using red and blue lines respectively. The lanes out of the lines is untreated HEK293T cells, which were not included in the main figure.

Supplementary Tables

Supplementary Table S1. Primers used in genotyping and RT-PCR

Screening of gene targeting	F1	GTCAGGCATGTGTGAAAACG
	R1	AATCACAGCCCATCAACTCC
	F2	CTATGAAAGGTTGGGCTTCG
	R2	GAAAAAGCCACTCTGAGGGA
Screening of PGK-Neo deletion	F3	TAGTTGCCAGCCATCTGTTG
	R3	ATCTCCATCCATGGTTTTGC
Probe labeling	Forward	CCCACAAATCTGGTCAATCC
	Reverse	GTGCTCCCTGTTGGTGAAAT
RT-PCR for F8	Forward based on exon 19	GCTGGGATGAGCACACTTTT
	Reverse based on exon 23	TCAACTCCATGCGAAGAGTG
RT-PCR for GAPDH	Forward	GGGGAGCCAAAAGGGTCATCATCT
	Reverse	GACGCCTGCTTCACCACCTTCTTG

Supplementary Table S2. Potential off-target sites predicted by the PROGNOS bioinformatic tool

Off-target site	Genome region	Closest gene	TALEN recognition sites		Mismatch, bp	Spacer length, bp
L1R1-OT-1	Intron	GPC6	CTCAGGCATTTAATTAAAA	TTGCCTCTGGAACCTTAA	5+2	30
L1R1-OT-2	Intergenic	GTSF1	TTAAAACATGCCCTTTAAA	TTGACTCCTTCCCTTTAAA	4+4	18
L1R1-OT-3	Intergenic	LINC00343	TTCAGGCATTCCCTTTAAA	TTCAATCATTCAATTTAAA	4+4	15
L1R1-OT-4	Intergenic	XYLT1	TTTCTGCTGTAATCTTAA	CTTCCACTTGAACCTTAG	4+3	21
L1R1-OT-5	Intergenic	AGTPBP1	TTTGCACTGGAACCTCAC	TTTCCCCTCCACCCTTAA	3+4	19
L1R1-OT-6	Intron	DGCR2	TCCCCACAGGGAATTTAA	TTTCCACTGGAGCCTTTA	6+2	21
L2R2-OT-1	Intergenic	VPS13C	TTTTAAATGACTAATTACA	ACATTAATCAGTAAATTTT	6+2	20
L2R2-OT-2	Intron	TMEM206	TTTATCGAGGAAATTCCAC	ACATTAATCAGTAAATTTT	3+6	16
L2R2-OT-3	Intergenic	SLITRK6	TTTTAAATGACTAATTACA	ACATTAATCAGTAAATTTT	2+6	10
L2R2-OT-4	Intergenic	SNHG5	TTTTAAATGACTAATTACA	ACATTAATCAGTAAATTTT	2+6	27
L2R2-OT-5	Intergenic	LRRC4C	TTTTAAATGACTAATTACA	ACATTAATCAGTAAATTTT	6+3	14
L2R2-OT-6	Intron	PPFIA2	TTTTAAATGACTAATTACA	CACCTTAAAGGAGCTATTT	2+6	29
L2R2-OT-7	Intergenic	MIR1973	TTTTAAATGACTAATTACA	ACATTAATCAGTAAATTTT	6+2	16

Off-target sites predicted by the PROGNOS bioinformatic tool. The mismatch is indicated by colored letters.

Supplementary Table S3. Primers used in TALEN off-target analysis

Site	Forward primer	Reverse primer	Expected product size, bp
L1R1-OT-1	TTCCTTCCCCAGACAGAGATTCC	GCAATGCACAAAAAGAGTGCAAATAGGTGCT	339
L1R1-OT-2	CCCTGGGCTATATCTTCAGAAGTG	CGCCCAGCCTCAGTTTTTAAGAGT	467
L1R1-OT-3	GCAGTCACGTTGAGCTATTAGGC	CCCCAAGACAATTGGCAGGTAG	348
L1R1-OT-4	ATGGTGGCTGGTGTGCAAGAG	AGCCCAGGAATTCAAGACCAACC	326
L1R1-OT-5	ATCTCACGTGCCACTGTGGG	CAGCTCATTCCTCTCCCTCTGG	358
L1R1-OT-6	CCCACACAAGATGTCAGAGTTCC	CTGTTGACCTGTCCTCAGGTTTAC	354
L2R2-OT-1	GGAGAGGAGTCCTTTGCATCCTT	GCTGCCTTAAGATGGCAAGTCTG	344
L2R2-OT-2	CGGGAGAGAGAGATGTGTGAC	GGCCAAGGCCATTGACTCTTGA	348
L2R2-OT-3	CTCATTTCCAGGTCAAGTTAAGGATGAGTC	CTAACCAGGGTCCAAATACGAGG	681
L2R2-OT-4	GAAGCTGGGTCACAAGAGGTTAAC	GATGGGTTGATGGGTGCAGC	461
L2R2-OT-5	GAAGCCTCACCATAATCACACCAC	GAGGGGAGACTGCATTTGTCTTTC	501
L2R2-OT-6	CCTTGACATGCTCCCATCCTTTTG	CTCTTAAACCTTGAGGTTTCTGTTTGAGGG	442
L2R2-OT-7	CAGCAATAAAATAGAAGGGCTTTAGCCATTCC	CTGGATGAGCCCCAACAAATGC	330

Supplementary Methods

Cell culture

HEK293T cells were maintained in DMEM (Life Technologies) supplemented with 10% fetal bovine serum (FBS, Life Technologies). OP9 cells were maintained on gelatin coated dishes in α-MEM (HyClone) supplemented with 20% FBS. Urine cells were maintained on gelatin (Millipore) coated plates in REGM (Lonza). iPSCs were cultured on feeder cells in DMEM/F12 supplemented with 20% knockout serum replacement, 2 mM L-glutamine, 1% non-essential amino acids, 0.1 mM β-mercaptoethanol, 100 unit/ml penicillin and 100 mg/ml streptomycin and 10 ng/ml basic fibroblast growth factor (bFGF) (ES medium, all from Life Technologies). Feeder cells were derived from day 13.5 CF-1 embryos and mitotically inactivated by mitomycin C (Calbiochem) treatment. Alternatively, iPSCs could be maintained on matrigel^{TM} (BD Biosciences) coated dishes in mTeSR 1 medium (stem cell technologies).

Karyotyping

After being treated with 0.08 μg/ml colcemid (Sigma-Aldrich) for 2-3 hours, cells were tripsinized, pelleted, and incubated in 0.075 M KCl for 30 minutes at 37°C. After being fixed with Carnoy fixative, metaphase chromosome spreads were prepared using air drying method. The chromosomes were G-banded with Giemsa Stain (Sigma-Aldrich) after appropriate baking at 75°C and digestion with trypsin.

Flow cytometry

The iPSCs and iPSCs derived MSCs were harvested using 0.25% Trypsin/EDTA.

After neutralization, cell suspensions were pelleted and washed in DPBS. Then the cells were resuspended in 3 ml DPBS and divided in to 300μL aliquots. Fluorescein isothiocyanate (FITC)-conjugated mAbs against human CD24(20μl), CD90(20μl) and phycoerythrin (PE)-conjugated mAbs against human CD34(20μl), CD44(20μl), CD45(20μl), CD73(20μl), CD31(20μl), CD105(5μl) were added into the aliquots respectively. (All from BD Pharmingen) After thirty minutes of incubation at room temperature, cells were washed with 5ml DPBS. Then the cells were resuspended in 500μl DPBS and analyzed with a FACSCalibur flow cytometer (BD Biosciences).

FVIII assay of culture supernatant and cell lysate

Twenty-four hours old culture supernatants were collected from 12-well plates. Then the cells were trypsinized and counted. After washed with PBS, the pelleted cells were resuspended in 500 μL sample diluent for ELISA (CEDARLANE) and lysed by 3 freeze-thaw cycles. All samples were collected in triplicate. ELISA was performed using Paired Antibodies for ELISA-Factor VIII:C (Cedarlane Laboratories) according to the manufacturer’s instructions. Reference curves were constructed using serial dilutions of normal pooled plasma, with correlation coefficient (R^2) of at least 0.980 using a semi-log fit. For FVIII activity assay, cell-free supernatant was collected twenty-four hours after medium change and concentrated 5-fold using a centrifugal filter (Millipore). Coagulation Factor VIII Deficient Plasma (Siemens) and Destiny Max™ Haemostasis Analyser (Tcoag) were used to examine the activated partial thrombo-plastin time (aPTT), for calculating the FVIII activity according to the manufacturer's instructions.
